# Supplementary material for: Lox’d in translation: contradictions in the nomenclature surrounding common lox-site mutants and their implications in experiments
Source: Microbiology (Reading). 2020 Dec 7;167(1):000997. doi: 10.1099/mic.0.000997 (PMC8116776; doi:10.1099/mic.0.000997)
Supplement: Supplementary material 1 [file mic-167-997-s001.pdf]

Suppl. Table 1: List of all plasmids used in this study.

Name	Description	Reference
pMTnCat	pMTn4001 containing a chloramphenicol resistance gene under the control of the 22 bp pM438 promoter from *Mycoplasma genitalium*.	(63)
pBSK_Cre_Sce_Puro	Modified pBSKII+ vector, containing the Cre recombinase, *I-SceI* meganuclease and puromycin resistance under the control of the pM438 promoter.	This study
pMTn_Lox66_Sce_Cm	Transposon derived from the pMTnCm plasmid, containing a lox66 site, *I-SceI* meganuclease recognition site and chloramphenicol resistance marker. Utilised the *Tn4001* transposase.<br>The pMTnCat vector was amplified by oligos 1 & 2, creating a 4992bp band, which was self annealed.	This study
pBSK_p438_Cre_Puro	Modified pBSK_Cre_Sce_Puro vector, containing just the Cre recombinase and puromycin resistance marker under the control of the pM438 promoter.<br>The pBSK_Cre_Sce_Puro vector was amplified with oligos 3 & 4, creating a 4646bp band, which was self annealed.	This study
pBSK_p438_Sce_Puro	Modified pBSK_Cre_Sce_Puro vector, containing just the *I-SceI* meganuclease and puromycin resistance marker under the control of the pM438 promoter.<br>The pBSK_Cre_Sce_Puro vector was amplified with oligos 5 & 6, creating a 4285bp band, which was self annealed.	This study
pBSK_p438_Puro	Modified pBSK_Cre_Sce_Puro vector, containing just a puromycin resistance marker under the control of the pM438 promoter.<br>The pBSK_Cre_Sce_Puro vector was amplified with oligos 7 & 6, creating a 4285bp band, which was self annealed.	This study

CONFIDENTIAL

Suppl. Table 2: List of oligos used in this study

Name	Sequence (5' to 3')

1	TACCGTTCGTATAATGTATGCTATACGAAGTTATTAGGGATAACAGGGTAATTAGTATTTAGAATTAATAAAGTATG
2	ATTACCCTGTTATCCCTAATAACTTCGTATAGCATACATTATACGAACGGTACGATATCAAGCTTATCGATACC
3	CCTGAAATAACGAATTCCTGC
4	CAGGAATTCGTTATTTCAGGCTAGAATTCGATCTAATCGCC
5	ACGGTATCGATAAGCTTGATAATTCTAGTATTTAGAATTAATAAAGTATGC
6	ATCAAGCTTATCGATACCG
7	ACGGTATCGATAAGCTTGATATAACGAATTCCTGCAGGG

Suppl. Table 3: CFU per 10µl for the Cre & *I-SceI* lethality tests

	WT_Cre	P0.1_Cre	WT_*I-SceI*	P0.1_*I-SceI*	WT_Blank	P0.1_Blank
Rep. 1	5.60E+05	8.00E+02	5.20E+05	2.50E+03	2.11E+08	1.38E+08
Rep. 2	6.10E+05	2.30E+03	4.80E+05	1.40E+03	1.87E+08	1.81E+08
Rep. 3	6.30E+05	1.70E+03	3.80E+05	1.70E+03	2.19E+08	1.60E+08

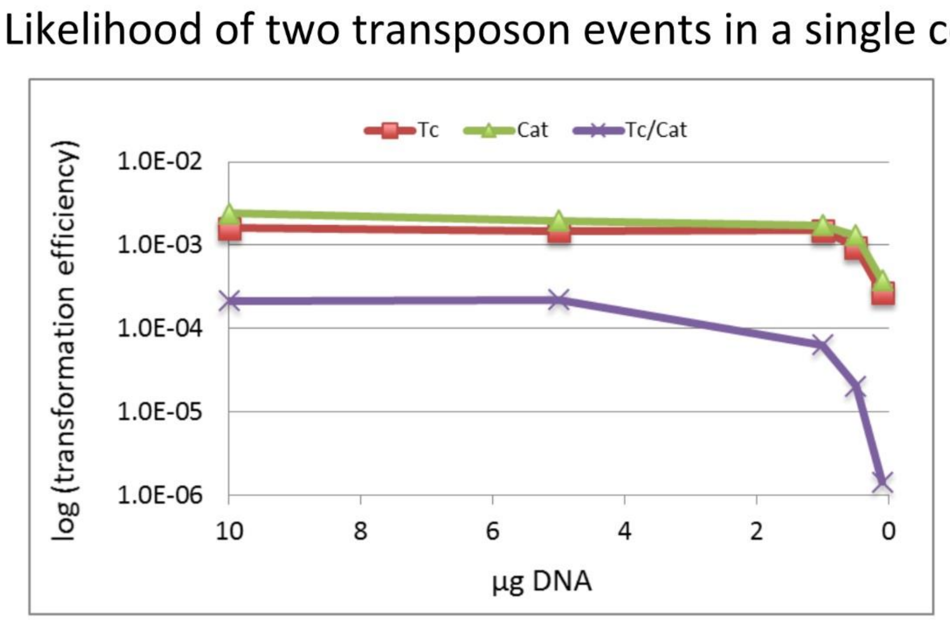

Suppl. Figure 1: Transformation efficiencies of *Tn4001* transposons in *M pneumoniae* when using differing volumes of DNA. The Cat and Tc conditions represent transformations with the pMTnCat and its tetracycline resistant variant pMTnTc respectively. Tc/Cat represents transformation with both plasmids concurrently. Transformation efficiency represents the proportion of cells that were successfully transformed.
